# Shaping pseudoneglect with transcranial cerebellar direct current stimulation and music listening

**DOI:** 10.3389/fnhum.2015.00158

**Published:** 2015-03-26

**Authors:** Silvia Picazio, Chiara Granata, Carlo Caltagirone, Laura Petrosini, Massimiliano Oliveri

**Affiliations:** ^1^Clinical and Behavioral Neurology Laboratory, Non-Invasive Brain Stimulation Unit, IRCCS “Santa Lucia” FoundationRome, Italy; ^2^Department of Psychology, “Sapienza” University of RomeRome, Italy; ^3^Department of Psychology, University of PalermoPalermo, Italy; ^4^Department of Neuroscience, “Tor Vergata” University of RomeRome, Italy; ^5^NeuroTeam Life and Science InstitutePalermo, Italy

**Keywords:** state dependency, cerebellum, music listening, tcDCS, pseudoneglect

## Abstract

Non-invasive brain stimulation modulates cortical excitability depending on the initial activation state of the structure being stimulated. Combination of cognitive with neurophysiological stimulations has been successfully employed to modulate responses of specific brain regions. The present research combined a neurophysiological pre-conditioning with a cognitive conditioning stimulation to modulate behavior. We applied this new state-dependency approach to investigate the cerebellar role in musical and spatial information processing, given that a link between musical perception and visuo-spatial abilities and a clear cerebellar involvement in music perception and visuo-spatial tasks have been reported. Cathodal, anodal or sham transcranial cerebellar Direct Current Stimulation (tcDCS) pre-conditioning was applied on the left cerebellar hemisphere followed by conditioning stimulation through music or white noise listening in a sample of healthy subjects performing a Line Bisection Task (LBT). The combination of the cathodal stimulation with music listening resulted in a marked attentional shift toward the right hemispace, compensating thus the natural leftward bias of the baseline condition (pseudoneglect). Conversely, the anodal or sham pre-conditioning stimulations combined with either music and white noise conditioning listening did not modulate spatial attention. The efficacy of the combined stimulation (cathodal pre-conditioning and music conditioning) and the absence of any effect of the single stimulations provide a strong support to the state-dependency theory. They propose that tcDCS in combination with music listening could act as a rehabilitative tool to improve cognitive functions in the presence of neglect or other spatial disorders.

## Introduction

The recent state-dependency theory proposes that the effects of an external stimulus on brain excitability depend not only on the properties of that particular stimulus but also on the activation state of the brain structures involved (Boly et al., 2007; Becker et al., 2008, 2011; Silvanto et al., 2008). State-dependency theory has been tested and validated under different experimental protocols and for multiple brain areas (Cattaneo et al., 2008, 2010). Indeed, the frequently observed variability in response across and within individuals following non-invasive brain stimulation may be related to the ongoing local neuronal activity the stimulus is encountering (Fox et al., 2007). For instance, conditioning excitatory trains of repetitive Transcranial Magnetic Stimulation (rTMS) over the motor cortex results in increased or decreased excitability, depending on inhibitory or facilitatory pre-conditioning (by transcranial Direct Current Stimulation, tDCS) of the same area (Lang et al., 2004). The well-known paradigms of adaptation and priming are the best examples of how the impact of an external stimulus depends on the state of the perceiver. The combination of pre-conditioning and conditioning brain stimulations determines the ultimate response of the stimulated region. The state-dependency approach has been mainly applied by using neurophysiological brain stimulations for both pre-conditioning and conditioning of brain excitability (tDCS and rTMS) (Lang et al., 2004; Siebner et al., 2004). More recently, the activation states of distinct neural regions have been functionally (and not neurophysiologically) pre-conditioned prior to the application of a subsequent neurophysiological conditioning stimulation (by rTMS, TMS, tDCS, …) (Cattaneo et al., 2008, 2010; Weisz et al., 2012; Feurra et al., 2013). Indeed, sensory (differently filtered sounds) or cognitive (visual priming, motor imagery, observation of motor behavior) stimulations have been used as pre-conditioning tools (Cattaneo et al., 2008, 2010; Weisz et al., 2012).

In the present research we propose that the state-dependency theory could also allow inversely combining a neurophysiological pre-conditioning with a subsequent cognitive conditioning to modulate behavior. Furthermore, for the first time we apply the state-dependency approach to the study of cerebellar excitability. Indeed, it was previously shown that down-regulation of left cerebellar hemisphere excitability by continuous theta burst stimulation (cTBS) modulates mental rotation performance only when the task was executed during music listening and not in silence (Picazio et al., 2013a,b). It was hypothesized that cerebellar pre-conditioning had primed neuronal populations facilitating the effects of subsequent music listening on the mental rotation task. However, such an interpretation could only be proposed and not demonstrated because of the features of the experimental protocol (lack of different kinds of pre-conditioning, conditioning stimulation and task performance occurring at the same time). Thus, the present research investigates the effects of cerebellar neurophysiological pre-conditioning (by transcranial cerebellar Direct Current Stimulation, tcDCS) followed by a cognitive conditioning procedure (music listening) in a sample of healthy musically naïve subjects performing a Line Bisection Task (LBT).

LBT was chosen because it enables a detailed analysis of any leftward or rightward shift that is distinct from mental rotation. LBT tests spatial representations for accuracy, by evaluating the distance between bisection point (transector) and veridical midpoint. It has been widely reported that patients with neglect show an impaired LBT performance due to a strong overestimation bias towards the right part of the space, with a decrease of accuracy and precision leading to increased variability. On the contrary, healthy subjects typically show a systematic preference towards the left hemispace defined as pseudoneglect (Bowers and Heilman, 1980; McCourt, 2001; Brooks et al., 2014). Pseudoneglect is probably due to the supremacy of the attentional vector leading the attention towards the left hemifield (Reuter-Lorenz et al., 1990; Fink et al., 2000; Fierro et al., 2001).

TDCS has already been successfully utilized to modulate visuo-spatial attention in healthy subjects by targeting the parietal lobes (Bowers and Heilman, 1980; Sparing et al., 2009; Giglia et al., 2011; Loftus and Nicholls, 2012). Namely, it was demonstrated that both anodal tDCS over the left posterior parietal cortex and cathodal tDCS over the right homolog parietal cortex are able to induce a rightward attentional shift and compensate pseudoneglect (Giglia et al., 2011; Loftus and Nicholls, 2012).

**Figure 1:**
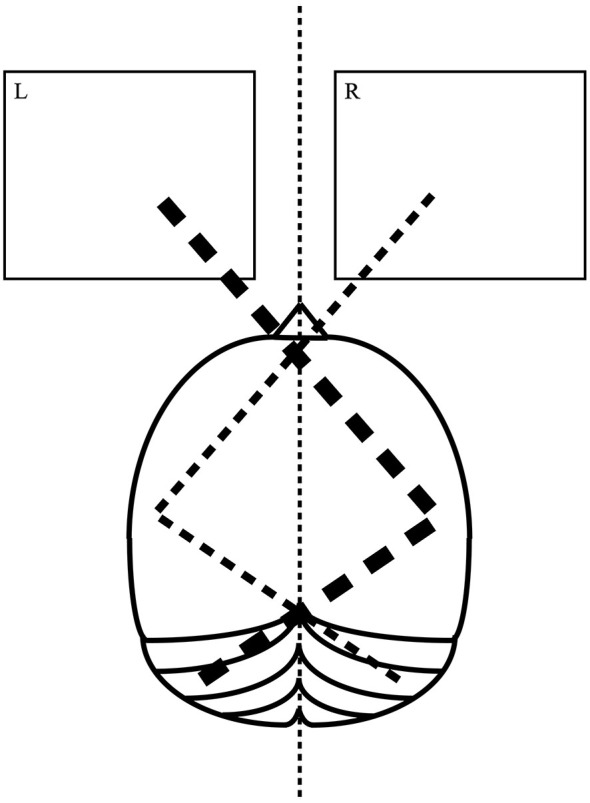
**A model of cerebellar involvement in visuo-spatial lateralized attention**. Left cerebellar hemisphere works in network with the right cerebral hemisphere for directing the attention toward the left visual hemifield. Right cerebellar hemisphere works in network with the left cerebral hemisphere for directing the attention toward the right visual hemifield. The attentional vector for the left visual hemifield (thick dashed line) is naturally stronger than the one for the right visual hemifield (thin dashed line).

In the present study for the first time tDCS was applied over a cerebellar site to modify pseudoneglect. The involvement of the left cerebellar hemisphere in visuo-spatial attention and in LBT has been consistently described in neuroimaging (Fink et al., 2000), brain stimulation (Oliver et al., 2011) and neuropsychological (Daini et al., 2008) studies. According to the model of crossed cerebello-cortical interactions, the cerebellar hemispheres and contralateral cortical regions are interconnected via thalamus (Clower et al., 2001; Middleton and Strick, 2001; Dum and Strick, 2003). As represented in Figure 1, left cerebellar hemisphere and right parietal lobe work together for directing the attention toward the left visual hemifield with greater strength than the contralateral attentional vector.

The use of LBT paradigm was further motivated by previous research showing that visual spatial attention is modulated by auditory cues (Patston et al., 2006; Tillmann et al., 2010; Ishihara et al., 2013; Lega et al., 2014).

Music listening was chosen as conditioning procedure since it probably represents an excitatory input for the left cerebellar hemisphere, as indicated by the marked left cerebellar activations during several auditory tasks (Petacchi et al., 2005). Evidence for the cerebellar involvement in melodic, rhythmic and timbral processing during music listening has been repeatedly provided (Griffiths et al., 1999; Gaab et al., 2003; Alluri et al., 2012; Toiviainen et al., 2013).

The link between music listening and spatial abilities has been previously described (Stewart and Walsh, 2007; Cattaneo et al., 2014; Lega et al., 2014). In musically untrained subjects, listening to sounds of different pitch prompts mental spatial representations (Rusconi et al., 2006; Lidji et al., 2007). Tone-deafness commonly referred as amusia is frequently correlated with deficits of spatial processing (Douglas and Bilkey, 2007; Williamson et al., 2011). Visuo-spatial perception and imagery abilities are enriched in musicians compared with nonmusicians as indicated by the shorter reaction times musicians exhibit (Brochard et al., 2004). Neurobehavioral evidence supports the transferable benefit of music training to enhance cognitive performance in visuo-spatial tasks. Furthermore, functional magnetic resonance imaging revealed that only musicians show increased activation in Broca’s area, in addition to the typical activation of the visuo-spatial network (Sluming et al., 2007). Taken together, these data suggest that musical processing uses spatial representations at least partially, and that in turn musical processing can enhance visuo-spatial abilities (Rauscher et al., 1993, 1995; Stewart and Walsh, 2007). The chance of a functional interdependence between musical and visuo-spatial domains motivates the choice to harness music processing to modify spatial attention in the present state-dependency study. To investigate the effects of combined neurophysiological cerebellar pre-conditioning and music listening conditioning, as conditioning stimulation we chose the Mozart Sonata K. 448 since it has been repeatedly employed in studies on the association between musical and spatial abilities (Rauscher et al., 1993, 1995; Bodner et al., 2001). As a control conditioning stimulation we chose White Noise listening to provide a baseline measure for neutral auditory stimulation (Ishihara et al., 2013; Lega et al., 2014). A group of healthy musically naïve subjects executed the LBT before and after (cathodal, anodal or sham tcDCS) pre-conditioning stimulation on the left cerebellar hemisphere followed by an auditory conditioning stimulation (Mozart Sonata or White Noise listening).

On the basis of state-dependency theory, baseline performance (pseudoneglect) should change only following combined pre-conditioning and conditioning stimulations. In fact, the single effects of music listening and neurophysiological could be too weak for example because of the time spent listening to a neutral stimulus, as white noise. Following the combined anodal tcDCS + Music or cathodal tcDCS + Music stimulations a polarity-dependent effect on LBT performance was expected.

## Materials and Methods

### Participants

Thirteen healthy subjects (7 women: 23.6 ± 2.2 years; schooling >10 years) were enrolled in this cross over study. All participants were right-handed as assessed with the Edinburgh Handedness Inventory (Oldfield, 1971) and reported normal- or corrected-to-normal vision and no hearing problems. None of the participants was musician or even non-professional player of a musical instrument as investigated with a brief telephone interview at the time of recruitment. Furthermore, no included person declared to be an enthusiast or an expert on classical music. The study was in accordance with the declaration of Helsinki and was approved by the Local Ethics Committee of the IRCCS “Santa Lucia” Foundation of Rome.

Written consent was obtained from all participants after a full explanation of the procedures of the study. At the end of the experiment the subjects were remunerated.

### Line Bisection Task (LBT)

**Figure 2:**
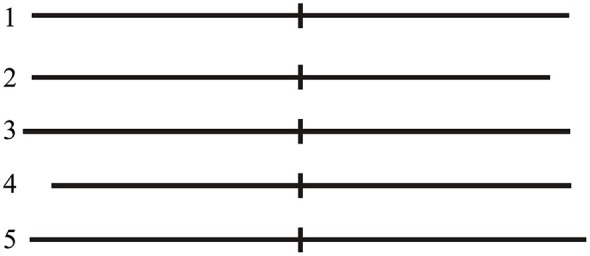
**LBT stimuli. Line 1** (exactly bisected): left segment 75 mm; right segment 75 mm. **Line 2** (left-elongated): left segment 75 mm; right segment 70 mm. **Line 3** (left-elongated): left segment 80 mm; right segment 75 mm. **Line 4** (right-elongated): left segment 70 mm; right segment 75 mm. **Line 5** (right-elongated): left segment 75 mm; right segment 80 mm.

The present task was a modified version based on the protocol of another study published by Fierro et al. (2001). Participants were tested in a quiet room of our lab. They sat comfortably on an armchair at a distance of about 100 cm from a computer monitor; the center of the monitor was aligned with the subject’s eyes. A computerized version of LBT was used. Stimuli consisted of a single black 1 mm thick horizontal line transected by a 1 mm thick and 10 mm high vertical bar (transector), presented on a white background with the transector exactly coincident with the center of the screen. The stimuli differed in the overall line length and in the position of the transector (exactly at midpoint, displaced of 5 mm rightward or leftward) determining slightly different lengths of the right and left segments of the line (Figure 2). Stimuli were tachistoscopically presented (50 ms duration) to prevent eye scanning. Before stimulus presentation, the subject was required to fixate for 250 ms a circular central target on the blank screen that disappeared as soon as the visual stimulus was presented. After presentation of each stimulus, the subject was required to indicate the longer line segment, making a forced-choice decision with three response possibilities (equal segments, longer to the right, or longer to the left). Responses were made by pressing with the right index finger the central, the right or the left key of a three key-button box to indicate respectively that the two segments were of equal length, that the right segment was longer, that the left segment was longer. Each LBT block involved 60 randomized trials including 20 repetitions of exactly bisected lines (Line 1), and 10 repetitions of right- or left-elongated lines (Lines 2–5, see Figure 2). Participants were encouraged to complete the whole LBT task and to respond even when doubtful.

Subjects’ performance on each trial was arbitrarily scored as described by Fierro et al. (2001). Namely, accuracy on each trial was scored as follows: 0 = correct response; 1 and 2 = rightward error due to left under-evaluation (1 = right segment of line 1 judged longer, or left and right segments of lines 2 and 3 judged equal; 2 = right segment of lines 2 and 3 judged longer); −1 and −2 = leftward errors due to right under-evaluation (−1 = left segment of line 1 judged longer, or left and right segments of lines 4 and 5 judged equal; −2 = left segment of lines 4 and 5 judged longer).

For each experimental condition the total score was obtained by averaging the sixty responses of each block. Mean negative values indicated a leftward bias (right under-evaluation), while positive values indicated a relative rightward bias (left under-evaluation) (Fierro et al., 2001; Ribolsi et al., 2013).

### Transcranial Cerebellar Direct Current Stimulation

The tcDCS was applied over the left cerebellar hemisphere through two sponge electrodes (surface area = 25 cm^2^) moistened with a saline solution. One electrode was centered on the left cerebellar cortex, 1 cm under, and 3 cm laterally to the inion (approximately comparable to the projection of cerebellar lobule VII onto the scalp). The second electrode was positioned on the left deltoid muscle (Ferrucci et al., 2008). The safety of using an extracephalic reference electrode for tDCS in healthy volunteers and that it doesn’t induce appreciable modulation of the brainstem autonomic centers has already been demonstrated (Vandermeeren et al., 2010). The onset and offset of all stimulations (anodal, cathodal, or sham) involved current being increased and decreased, respectively, in a ramp-like manner over 45 s as done in previous studies (Nitsche et al., 2003; Hummel et al., 2005). Stimulation was applied with intensity of 2 mA and delivered for 20 min by using a BrainStim Stimulator (EMS, Italy). This kind of stimulation provides a safe level of exposure well below the threshold for causing tissue damage (Boggio et al., 2006).

Sham stimulation consisted of cathodal tcDCS applied for only 45 s over the left cerebellar hemisphere and then turned off. This procedure was adopted to give the subject the same initial itching sensation of real tDCS stimulation but without receiving current as done in previous reports that employed tDCS to study cerebellar functions (Ferrucci et al., 2008; Galea et al., 2009). Participants were unaware of the features of the stimulations.

### Musical/White Noise Listening

Participants listened to the Sonata in D Major K. 448 for two pianos and orchestra by Mozart. In the control conditions, participants listened to a registration of randomly combined sounds of all audible frequencies (white noise). Listening was done through Philips stereo earphones. Both auditory stimuli lasting 11′ 28″ and volume were set at a high but comfortable level for all subjects (the same for both stimuli). Both music and white noise have a wide frequency range, stimulating a large number of hair cells in the cochlea and hence a large portion of the cortical auditory areas, but while white noise has a continuous and uniform frequency spectrum, the music is a stimulus with pitch, duration, intensity and timbre dynamically composed. At the end of the experiment in response to explicit questions of the investigator to record subjective reports during music or white noise listening, no participant reported to be bored or bothered by the Mozart Sonata listening, while sometimes they reported to be a little annoyed by listening to white noise.

### Experimental Procedure

Each subject was submitted in a counterbalanced order to six experimental sessions spaced by one week from each other. In each session, a single (anodal, cathodal or sham) tcDCS and listening (Mozart Sonata or white noise) were applied.

There were six experimental conditions involving a *pre* and a *post* stimulation: Sham pre-conditioning followed by White Noise listening: S + WNpre, S + WNpost; Sham pre-conditioning followed by Music listening: S + Mpre, S + Mpost; Cathodal pre-conditioning followed by White Noise listening: C + WNpre, C + WNpost; Cathodal pre-conditioning followed by Music listening: C + Mpre, C + Mpost; Anodal pre-conditioning followed by White Noise listening: A + WNpre, A + WNpost; anodal pre-conditioning followed by Music listening: A + Mpre, A + Mpost.

Before performing the real experiment, participants were presented with a practice session (results not included in the analyses), in which they were trained on six trials featuring lines of different lengths. No feedback on performance was given to the participants during the testing.

Afterwards, participants were submitted to the 60 trial-LBT. Pre-conditioning was performed by applying anodal, cathodal or sham tcDCS. Then, the subjects listened to either the Mozart Sonata or white noise for 11′ 28″ and immediately after, they were required to perform again 60 trials of LBT. Therefore, subjects did not execute any task during auditory stimulation and they were instructed to stay in silence in front of the computer monitor in the same position adopted during other experimental phases. This rest period could have potentially reduced the effect of the stimulation but the present study was designed to test an inversely combined state-dependency approach and therefore the combined effects of a neurophysiological pre-conditioning followed by a cognitive conditioning on the final behavioral output.

### Statistical Analyses

Data presented as mean ± SEM were tested for normality (Will-Shapiro’s test) and homoschedasticity (Levene’s test). A one-way ANOVA with repeated measures was performed on pre-values recorded before the six combined stimulation conditions to assess that task baseline performances were comparable. Then, a three-way ANOVA with repeated measure was computed considering Stimulation (Anodal *vs*. Cathodal *vs*. Sham), Listening (Music *vs*. White Noise) and Time (pre *vs*. post) as within-subjects factors on LBT values.

Duncan’s *post-hoc* comparisons were performed when necessary. Effect size was indicated as partial eta square. The level of significance was set at *p* < 0.05. Statistical evaluations were performed with Statistica 8.

## Results

All subjects were able to perform the LBT in all six conditions. Subjects did not complain of any harmful effects of tcDCS. The mean LBT performances of the 13 participants before and after the six stimulations are shown in Table 1.

**Table 1:** **LBT performances before (pre) and following (post) neurophysiological and cognitive stimulations**.

	Conditions
	S + WN	S + M	C + WN	C + M	A + WN	A + M
Pre	−0.00 ± 0.17	−0.04 ± 0.15	−0.04 ± 0.12	−0.10 ± 0.14	−0.07 ± 0.22	−0.02 ± 0.16
Post	−0.02 ± 0.18	−0.06 ± 0.17	−0.08 ± 0.16	0.01 ± 0.16	−0.05 ± 0.13	−0.03 ± 0.19

*Values are represented as mean scores ± standard deviation (N = 13). S + WN: Sham tcDCS + White Noise listening; S + M: Sham tcDCS + Music listening; C + WN: Cathodal tcDCS + White Noise listening; C + M: Cathodal tcDCS + Music listening; A + WN: Anodal tcDCS + White Noise listening; A + M: Anodal preconditioning followed by Music listening*.

**Figure 3:**
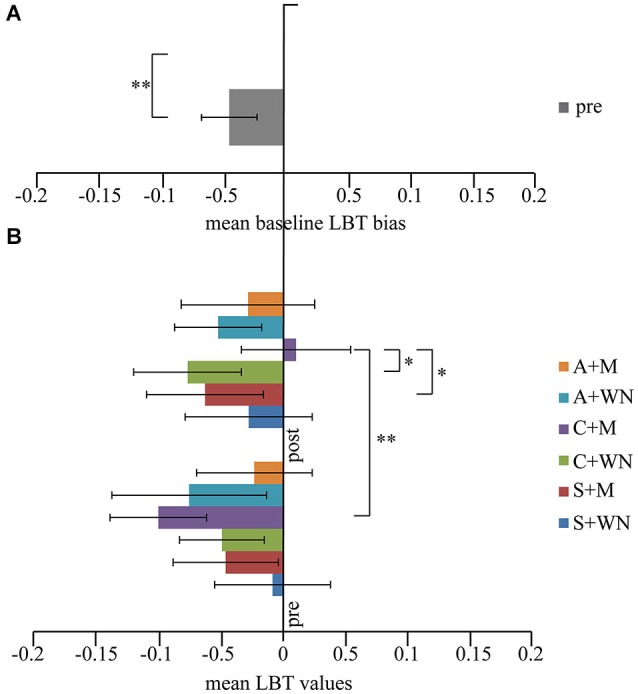
**(A) Mean pre values**. A slight spatial leftward (negative values) bias was observed. **(B) LBT performance**. Following Cathodal + M stimulation a significant shift toward the right space (positive values) was observed. *p < 0.05; **p < 0.01. Error bars indicated standard error of mean.

One-way repeated measure ANOVA on the scores obtained before the six combined stimulations revealed no significant difference (*F*_5,60_ = 1.17; *p* = 0.331). On average, the subjects judged the left segment of the line as longer than the right one, evidencing a slight spatial leftwards bias (pseudoneglect). By comparing the mean score ($\bar{\text{X}}$ : −0.047 ± 0.08) in all pre-stimulation conditions (baseline) with the theoretically unbiased score of 0, by means of a paired *T*-Test, a significant difference was found (*p* < 0.006) (Figure 3A).

Three-way repeated measure ANOVA (Stimulation × Listening × Time) showed no significant Stimulation [*F*_2,24_ = 0.19; *p* = 0.832; $\eta_{2}^{p}$ = 0.015], Listening [*F*_1,12_ = 0.18; *p* = 0.679; $\eta_{2}^{p}$ = 0.015] and Time [*F*_1,12_ = 0.09; *p* = 0.758; $\eta_{2}^{p}$ = 0.008] effects but significant Listening × Time [*F*_1,12_ = 6.91; *p* = 0.022; $\eta_{2}^{p}$ = 0.365] and Stimulation × Listening × Time [*F*_2,24_ = 3.73; *p* = 0.039; $\eta_{2}^{p}$ = 0.237] interactions. Stimulation × Listening [*F*_2,24_ = 0.69; *p* = 0.512; $\eta_{2}^{p}$ = 0.054] and Stimulation × Time [*F*_2,24_ = 2.49; *p* = 0.104; $\eta_{2}^{p}$ = 0.172] interactions did not reach significance level. *Post-hoc* comparisons on the Stimulation × Listening × Time interaction revealed that the C + Mpost condition was significantly different from C + Mpre (*p* = 0.002), S + Mpost (*p* = 0.048) and C + WNpost (*p* = 0.049) conditions. In C + Mpost condition a performance closer to the real center was found, while all remaining conditions evoked a leftward bias. Within the significant differences between C + Mpost and the other conditions, it is worth noting the significant difference between C + Mpost and C + WNpost (*p* = 0.049), indicating the absence of any effect of cathodal stimulation *per se*. No significant differences were found between S + WNpost and S + Mpost conditions (*p* = 0.255), indicating the absence of a music listening effect *per se* on LBT. Furthermore, no significant difference was found among the three white noise conditions (A + WNpost *vs*. C + WNpost − *p* = 0.631; S + WNpost* vs*. A + WNpost − *p* = 0.455; S + WNpost* vs*. C + WNpost − *p* = 0.243), indicating the absence of WN conditioning effect on LBT (Figure 3B).

## Discussion

The present results indicate that not musically trained healthy subjects bisect lines with a leftward bias, showing the previously described pseudoneglect (Bowers and Heilman, 1980). The pseudoneglect implies a mild, spontaneous preference for left hemispace related to a dominance of the right cerebral hemisphere in spatial judgments (Corbetta et al., 1993) that extends to the mental representation of space (Oliveri et al., 2004; Brooks et al., 2014).

Our results demonstrate that the cerebellar activation state efficiently influenced the complex auditory (music) and visuo-spatial (LBT) processing. Namely, the combination of the cathodal cerebellar pre-conditioning with music conditioning (but not the sham or anodal pre-conditioning combined with either music or white noise conditioning) provoked a shift toward the right hemispace, resulting in a more symmetrical distribution of attention. The rightward shift compensated the natural bias to be docked to the left hemispace and approached the fully centered LBT response. This finding nicely fits with the rightward shift in number line bisection task recently described following left cerebellar down-regulation (Oliver et al., 2011). The comparison between C + Mpost and C + WNpost conditions demonstrates the efficacy in combining a neurophysiological pre-conditioning with a cognitive conditioning, an inverse combination in respect to the previously performed cognitive pre-conditioning with a subsequent neurophysiological conditioning (Cattaneo et al., 2008, 2010; Weisz et al., 2012) and confirms the usefulness of employing transcranial direct current stimulation as useful tool to elicit state-dependency effects (Lang et al., 2004; Siebner et al., 2004; Feurra et al., 2013). Thus, we evidenced that *the “same” activation state of a given neuronal circuit* (the down-regulation of the left cerebellar hemisphere) *produces different effects on the task depending on the cognitive conditioning stimulation* (music or white noise).

A further point to be addressed is the tcDCS polarity-specificity. While some authors (Ferrucci et al., 2008, 2012; Shah et al., 2013) described polarity-independent tcDCS effects on cognitive and emotional processing, other authors found clear polarity-specific effects on cerebello-brain inhibition (Galea et al., 2009, 2011; Jayaram et al., 2012; Chen et al., 2014). In analogy with the results reported by Pope and Miall (2012) on working memory and attentive functions, in the present study we found a modulation of LBT performances only after cathodal stimulation, while sham and anodal stimulations did not modulate the pseudoneglect.

Recent studies have indicated that musical experience can shape spatial attention and pseudoneglect. Namely, musicians bisected lines more accurately and with an opposite (rightward) bias with respect to non-musicians (Patston et al., 2006). Moreover, low pitch listening produced leftward bias, whereas high pitch listening produced rightward bias both in musicians (Lega et al., 2014) and non-musicians (Ishihara et al., 2013). Consistently with the idea that music listening can produce perceptual and cognitive effects beyond the auditory modality, a very recent study indicated that while deaf participants showed a tendency to deviate rightward, normally-hearing subjects displayed the typical leftward pseudoneglect (Cattaneo et al., 2014). The present results suggest that not only a long-lasting musical training (as occurring in musicians) or the absence of auditory perception (as occurring in deafness) modulate visuo-spatial attention, but also a few minutes of music listening (*as long as combined* with the appropriate cerebellar pre-conditioning) modulate spatial attention inducing a rightward deviation closer to the real center.

The results obtained with music listening might be due to unspecific pleasantness-arousal effects. In fact, while 11′ 28″ of Mozart Sonata listening were perceived by all participants as agreeable, the same time of white noise listening was sometimes perceived as a boring experience. However, the lack of any modulating effect of both S + WNpost and S + Mpost conditions makes this hypothesis unlikely. Indeed, if a music-induced pleasantness-arousal effect were present, then it should have occurred regardless of the type of neurophysiological manipulation applied over the cerebellum. It has already been suggested that decreased levels of alertness may induce a rightward shift in visuo-spatial attention in healthy subjects (Manly et al., 2005). We assume that few minutes of listening were not enough to change the level of arousal and thus visuo-spatial performance.

The present findings agree with the cerebellar activations described during rhythm (Chen et al., 2008), pitch (Gaab et al., 2003), and timbre (Alluri et al., 2012) processing. The absence of spatial effects in the white noise conditions (A + WN, C + WN, S + WN) could be explained considering that white noise does not require any processing of rhythm, pitch or timbre, but only listening to random acoustic signals.

The presence of a combined effect of cerebellar down-regulation and music listening (C + M) and the absence of any effect of music listening *per se* (S + M) on visuo-spatial processing provide a strong support to the state-dependency theory. We suggest that the music-induced activation of cerebellar circuits previously down-regulated by cathodal pre-conditioning resulted in an increased activity of left cerebellar cortex that in turn inhibited the excitability of the right parietal cortex. The enhanced inhibitory control on the right cerebral hemisphere in general and of right parietal cortex in particular undocked the spatial attention from the left hemispace and allowed a shift toward the right hemispace. Conversely, the “same” music-induced activation of cerebellar circuits previously up-regulated by anodal pre-conditioning resulted in a decreased activity of the left cerebellar cortex that in turn enhanced the excitability of the right parietal cortex keeping spatial attention docked to the left hemispace (pseudoneglect) (Figure 1). The present results suggest a possible cerebellar influence on parietal cortex activity and are consistent with previous findings showing a similar modulation of pseudoneglect by parietal tDCS. Namely, tDCS alters the interhemispherical balance between parietal cortices, compensating thus the pseudoneglect (Bowers and Heilman, 1980; Loftus and Nicholls, 2012). Furthermore, the down-regulation of the right parietal cortex excitability through cathodal tDCS induces a rightward shift in symmetry judgments (Sparing et al., 2009; Giglia et al., 2011). In the light of the Kinsbourne’s theory of interhemispherical competition, lowering the excitability of the dominant cortex in visuo-spatial attentional tasks produces an imbalance in favor of the contralateral hemisphere that can counteract the natural leftward bias.

As for the tcDCS action on cerebellar activity, it could alter the fine-tuning of membrane potential and relative pace-making properties of the only output of the cerebellar cortex represented by the Purkinje cells. This neuronal GABAergic population exerts a strong inhibitory control on contralateral cerebral cortex via the thalamic nuclei (Ferrucci and Priori, 2014). tcDCS pre-conditioning primes the wide cerebello-cortical (parietal) network by decreasing/increasing its excitability in accordance to the polarity (Liebetanz et al., 2002; Nitsche et al., 2008).

The effectiveness of synaptic processes is normally closely regulated to stabilize neuronal activity within a physiological range (Sejnowski, 1977). Making the amount of Long Term Potentiation (LTP) dependent on the level of activity in the postsynaptic neuron prevents the risk of triggering an uncontrolled increase in synaptic effectiveness (Turrigiano, 2008; Nahmani and Turrigiano, 2014). The greater the ongoing activity of the postsynaptic neurons, the less effective the processes leading to LTP, the more enhanced processes leading to Long Term Depression (LTD). Conversely, the lower the activity of the postsynaptic neurons, the more effective the processes leading to LTP. This is known as “homeostatic” plasticity (Bienenstock et al., 1982). As hypothesized by Siebner et al. (2004), the physiological mechanism of state-dependency might to be related to the homeostatic plasticity, so that the magnitude and direction of synaptic effects may be plastically adjusted according to the recent history of postsynaptic activity, maintaining it within distinct limits. Following the homestatic plasticity framework, the ongoing input effects (music listening) might depend on the hyper- or de-polarization of neurons (tcDCS-induced neuronal state) at that moment.

In conclusion, speculatively the state-dependency principle could be applied to the present results as follows: the tcDCS-induced change in postsynaptic activity of cerebellar neurons could cause a long-term modification threshold for synaptic plasticity induced by music listening. So, the decreased activity produced by cathodal tcDCS would favor the facilitatory effects of music listening on spatial task. In this sense, music neuro-modulation could interact with the activation state of the system, driven by tcDCS pre-conditioning, to shape the final result. However, further investigations on the state-dependency functions of the right cerebellar hemisphere are needed to confirm this interpretation.

Although the present research was not designed to provide therapeutic applications, it may have important implications for clinical studies. Music listening in combination with tcDCS could be usefully employed as rehabilitative tool to improve the brain functions in patients. In fact, exploiting on one hand pleasantness, easy availability and non-invasivity of music and on the other hand, the very low invasivity, painlessness, safety and portability of tcDCS, the use of such combined stimulations might be effective in the treatment of hemi-spatial neglect and other attentional/representative disorders.

## Author Contributions

MO and CC designed research; SP and CG performed research; SP and LP analyzed data; all authors discussed data; SP, LP and MO wrote the paper.

## Conflict of Interest Statement

The present research was conducted in the absence of any commercial or financial relationships that could be construed as a potential conflict of interest by all authors and institutions.
